# The Anionic Surfactant/Ionic Liquids Intercalated Reduced Graphene Oxide for High-performance Supercapacitors

**DOI:** 10.1186/s11671-018-2636-9

**Published:** 2018-07-20

**Authors:** Jun-Hong Lin

**Affiliations:** Department of Mold and Die Engineering, National Kaohsiung University of Science and Technology, Kaohsiung, Taiwan, Republic of China

**Keywords:** Supercapacitors, Reduced graphene oxide, Anionic surfactant, Interlayer distance

## Abstract

The thermally reduced graphene oxide (TRG) composites with various interlayer distances were synthesized. These TRG sheets are intercalated with anionic surfactant sodium dodecyl sulfate (SDS) to prevent the restacking between TRG sheets. A facile approach is employed to enlarge the interlayer distance between the TRG sheets by the Coulomb force interaction between the intercalated surfactants and the ionic liquids. A systematic investigation of the morphology and the electrical performances of these EDLC cells was carried out. It was found that the energy density of the cells is improved from 34.9 to 61.8 Wh/kg at 1 A/g suggesting that the increased interlayer distance could enlarge the accessible surface area for the ionic liquid electrolyte.

## Background

Supercapacitors have drawn much attention on researchers and applications especially in electric vehicles and portable devices because of their advantages of high-power density, long-cycle life, wide range of operating temperature, and almost maintenance free [1, 2]. It is known that supercapacitors store energy based on the ion adsorption at the interfaces between the electrodes and electrolytes, and thus called electrical double layer capacitor (EDLC). Since ion absorption requires large interface and the stored energy is related to *E* = 1/2CV^2^, the researchers on improving the performance of EDLC cells are mainly focusing on utilizing electrolytes with wide electric chemical windows, developing conductive materials with the large specific surface area, and tuning the electrode/electrolyte interface properties [3–6]. Ionic liquids(ILs), a special group of molten salts, are becoming favorable for EDLC electrolytes not only because of their wide electrochemical window (> 3 V) that can effectively enhance the energy density of the cells but also due to their advantages of low volatility and high stability at high temperature [7–10]. However, ILs comprise of both cations and anions. Also, the size of these comprising ions could be large depending on their compositions. Therefore, compared to the aqueous electrolytes with small ions, the large ion size of ILs might hinder the accessibility of small pores for ILs. On the other hand, toward the high specific surface area for ion storage, reduced graphene oxide (RGO) is promising because of its high electrical conductivity and large theoretical surface area (2630 m^2^ g^−1^) [11–14]. RGO can be derived from graphene oxide (GO) in a large scale since it can be obtained by the oxidation of natural graphite particles [12, 15]. During the oxidation process, the defects and hydrophilic oxygen groups are introduced to the hydrophobic basal plane of graphene resulting in the amphiphilic GO. Because of the attached oxygen functional groups, GO is an electrical insulator and can be dispersed as individual sheets to form a stable suspension in water [16]. Thermally reduced graphene (TRG) converts the GO to graphene powder at elevated temperatures. Without a commonly used strong chemical base, it is environmental friendly [13, 14]. However, without the oxygen groups, the hydrophobic nature of TRG is almost water insoluble thus hampering the further processing of material composites [16].

In the literature, ionic surfactants are often employed to stabilize RGO suspension in solution while preventing the restacking of the RGO sheets in solid [17, 18]. Ionic surfactants are amphiphilic compounds made up of ionic hydrophilic head groups and extended apolar, organic residues hydrophobic tails. Thus, the surfactant can interact with RGO through the Coulomb force between the charged head groups and the residual oxygen groups. Also, the hydrophobic interactions between the aliphatic chains and hydrophobic basal planes play a vital role in the stabilization of individual RGO sheets in water [17]. Zhang et al. [18] employed a series of ionic surfactants to stabilize the GO sheets during the reduction process. They found that the surfactants are successfully intercalated in both GO and RGO sheets to prevent the restacking phenomena. Also, their results indicate that for the same surfactant-intercalated electrode, it has a much larger capacitance in aqueous electrolytes than in ILs electrolytes. This might be due to the ion diameter of ILs that is usually large. For example, the average ion diameter of 1-ethyl-3-methylimidazolium bis(trifluoromethylsulfonyl)imide (EMI-TFSI) is *D* ~ 0.7 nm [19] which is larger than the reported interlayer distance of the surfactant-intercalated RGO (~ 0.4 nm). Thus, the small interlayer distance might prohibit the accessibility in between the RGO sheets for ILs electrolytes.

**Fig. 1:**
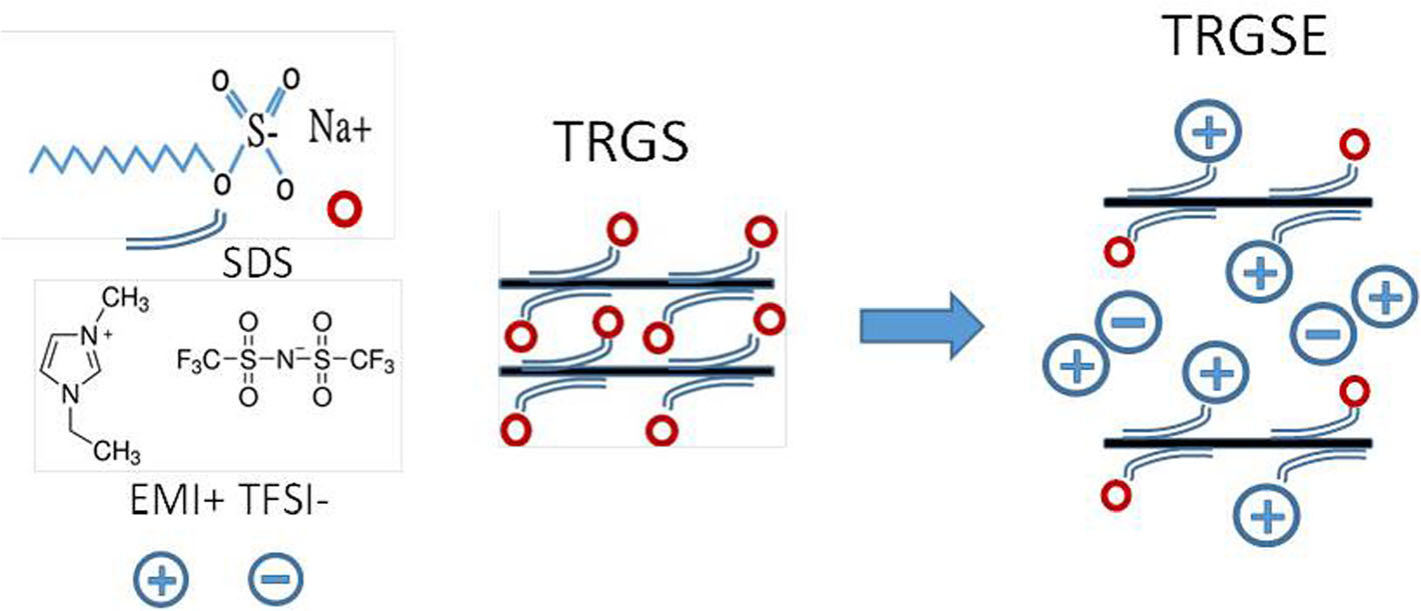
The schematic depicts the process to enlarge the interlayer distance between TRG sheets through the Coulomb force interaction

Here, we propose a facile method to enlarge the interlayer distance as shown in Fig. 1. The anionic surfactant sodium dodecyl sulfate (SDS) is intercalated in between TRG sheets forming a composite which was named as TRGS. Afterward, these intercalated TRGS sheets are rinsed with the EMI-TFSI solution during the filtration which was named as TRGSE. The Coulomb force among the intercalated ionic surfactants and the ILs might lead to the formation of ionic aggregates or micelles and thus increase the interlayer distance.

X-ray diffraction (XRD), small angle X-ray scattering (SAXS), Fourier-transform infrared spectroscopy (FTIR), and thermal gravimetric analysis (TGA) are employed to characterize the interlayer distance, the bond vibration, and the compositions of these electrode materials. Also, a VersaSTAT 4 potentiostat was used to characterize the electrical performance of these EDLC cells. It was found that the bond vibration representing the head group of the SDS was substantially changed due to the interaction with EMI-TFSI implying the Coulombic or ionic exchange interactions between SDS and EMI-TFSI. Furthermore, the X-ray results revealed an interlayer distance of 0.66 nm for the TRGS which proved the successful intercalation of SDS in between the TRGS sheets. Also, the interlayer distance of the TRGSE sheets is further enlarged to 3.92 nm suggesting the formation of SDS/EMI-TFSI aggregates or micelles. The substantial improvements in both capacitance and energy density of the TRGSE cells may mainly attribute to the large interlayer distance that leads to the more accessible surface area for the large-sized ILs.

## Results and Discussion

**Fig. 2:**
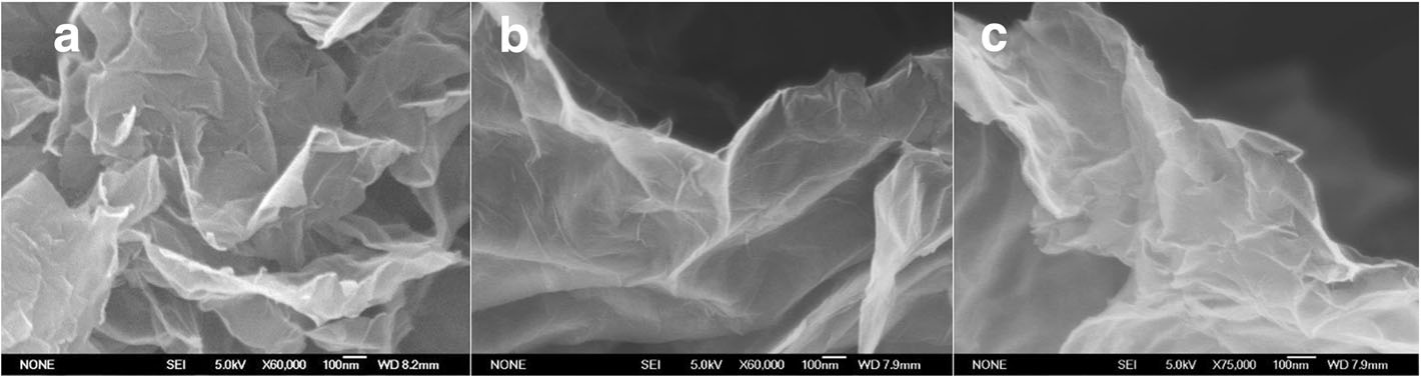
The SEM image of **a** TRG, **b** TRGS, and **c** TRGSE

The morphology of the TRG, TRGS, and TRGSE is characterized by the SEM. As shown in Fig. 2a, without SDS intercalation, the TRG would tend to agglomerate into graphite-like big particles. In contrast, in Fig. 2b,c, the SDS-stabilized TRGS and TRGSE seem to appear a more curved crumbly main structure with more irregular wrinkled edges implying the intercalation of the SDS that efficiently separate the TRG in a few layers of structure.

**Fig. 3:**
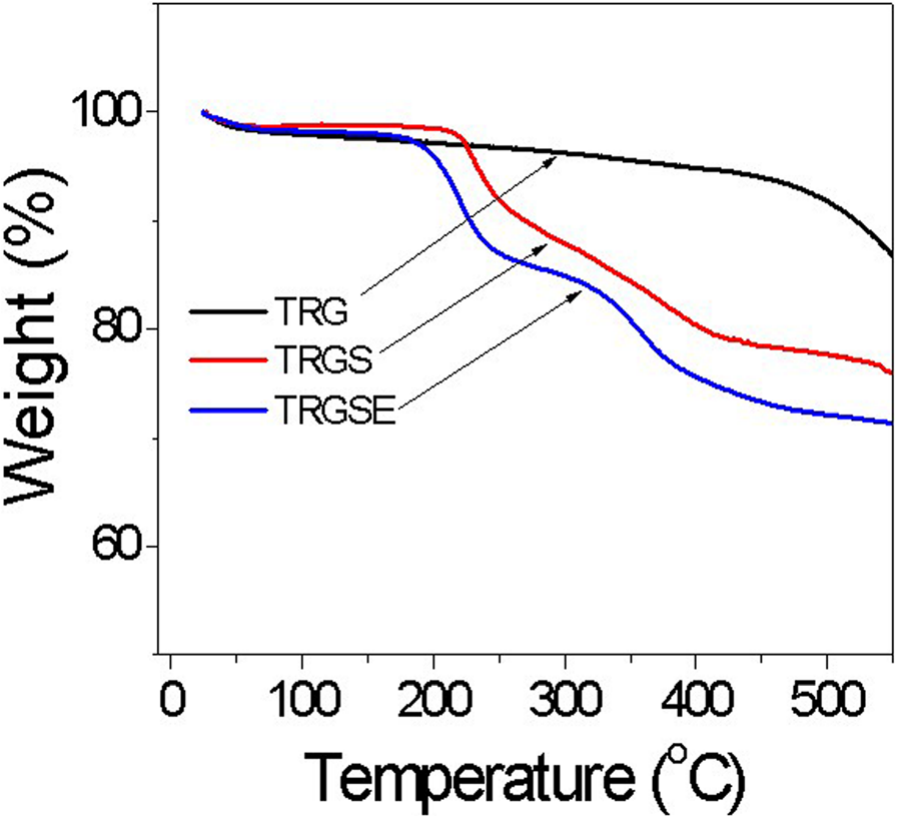
The thermal gravimetric analysis for TRG, TRGS, and TRGSE

Figure 3 plots the thermal gravimetric analysis results of the TRG, TRGS, and TRGSE composites. As observed, all the composites show a little mass loss around 100 °C due to the moisture content. The TRG displays a smooth weight loss from 100 to 500 °C with an 8% total weight loss at 500 °C followed by a steep weight loss due to the quick decomposition of the TRG. On the other hand, the TRGS and TRGSE show steep weight loss at around 200 °C which is ascribed to the decomposition of SDS and EMI-TFSI in thermally reduced graphene oxide sheets. The weight losses of 24 and 28% at 500 °C are observed for the TRGS and TRGSE samples, respectively. Compared with the TRGS, the weight loss of TRGSE is higher because of the participation of EMI-TFSI.

**Fig. 4:**
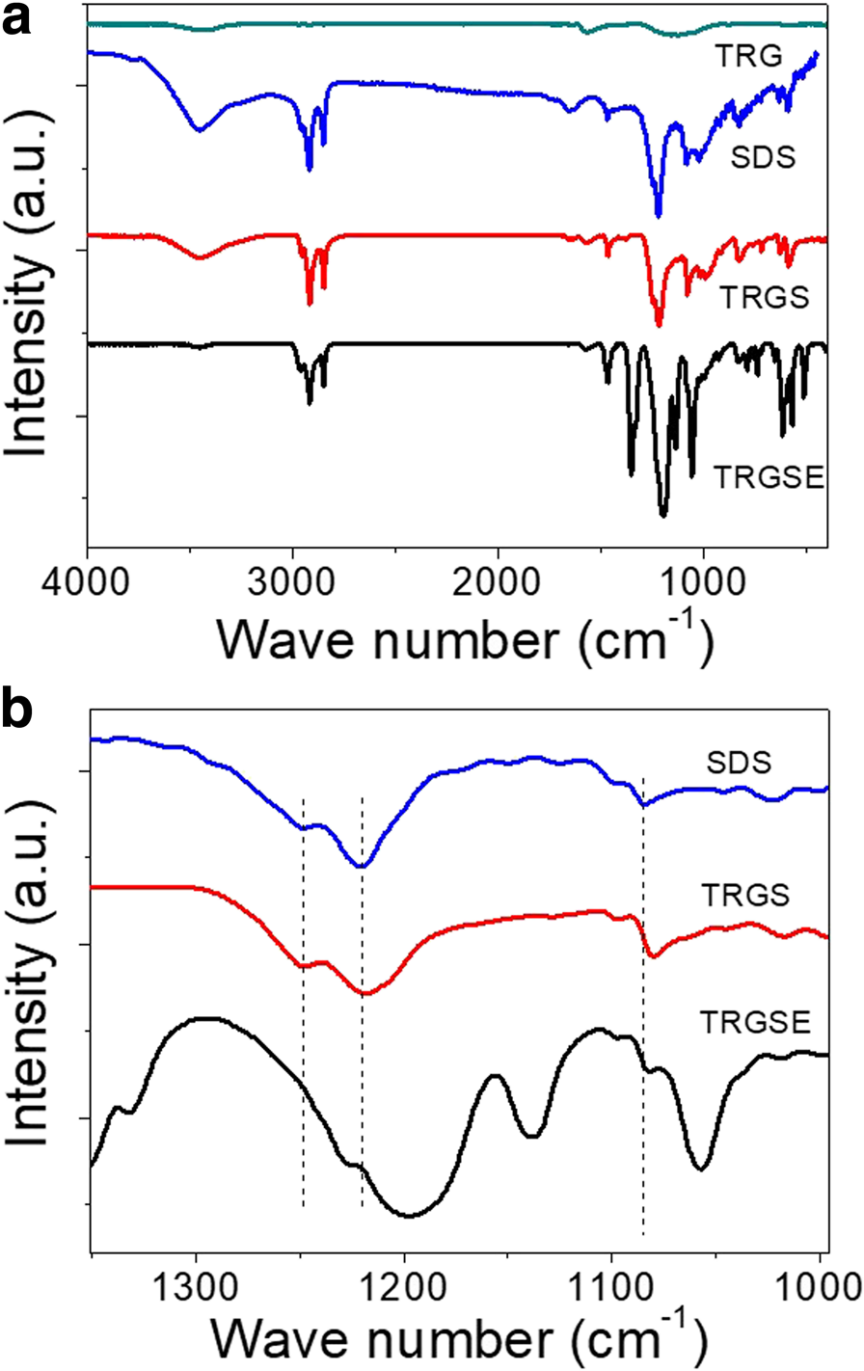
**a** FTIR spectra of the TRG, SDS, TRGS, and TRGSE. (**b**) Zoom in of **a** for a specific wavenumber region

Figure 4 plots the Fourier-transform infrared (FTIR) spectroscopy for neat TRG, neat SDS, TRGS, and TRGSE in the wavenumber region of (4000–400 cm^−1^). Several characteristic vibration modes of GO corresponding to its characteristic functional groups are often reported, including those in a higher frequency region, the bands at 3430, 1716, and 1635 cm^−1^ are corresponding to the stretching mode of O–H, C=O, and C=C, respectively. While that in a lower frequency region, the bands at 1033 and 1154 cm^−1^ are representing the stretching modes of C–O and C–OH, respectively. As shown in Fig. 4a, for the TRG sample, most of the oxygen-related groups are substantially removed leaving two small broad peaks at 1164 and 3430 cm^−1^ which corresponding to the residual C–OH and O–H groups. In Fig. 4a for the neat SDS, the band at 2955, 2917, and 2849 cm^−1^ are related to the C–H bond vibration [20, 21]. It seems that these C–H bands are not affected by both the TRG and EMI-FSI in the spectra of TRGS and TRGSE samples. Further, in Fig. 4b, the band at 1084 cm^−1^ representing the SO_2_ symmetric vibration for the neat SDS is found shifted to 1080 cm^−1^ in both TRGS and TRGSE spectra implying the interaction between the SDS surfactant and the TRG sheets. Also, in the TRGS spectrum (in Fig. 4b), the bands at 1219 and 1249 cm^− 1^ corresponding to the SO_2_ asymmetric vibration of neat SDS are not affected when the SDS is intercalated on to TRG [20, 21]. While in the TRGSE spectrum, the bands at 1219 and 1249 cm^−1^ are shifted to 1195 and 1226 cm^−1^, respectively. These shifts might be mainly a result of the interaction between the SDS and EMI-TFSI.

**Fig. 5:**
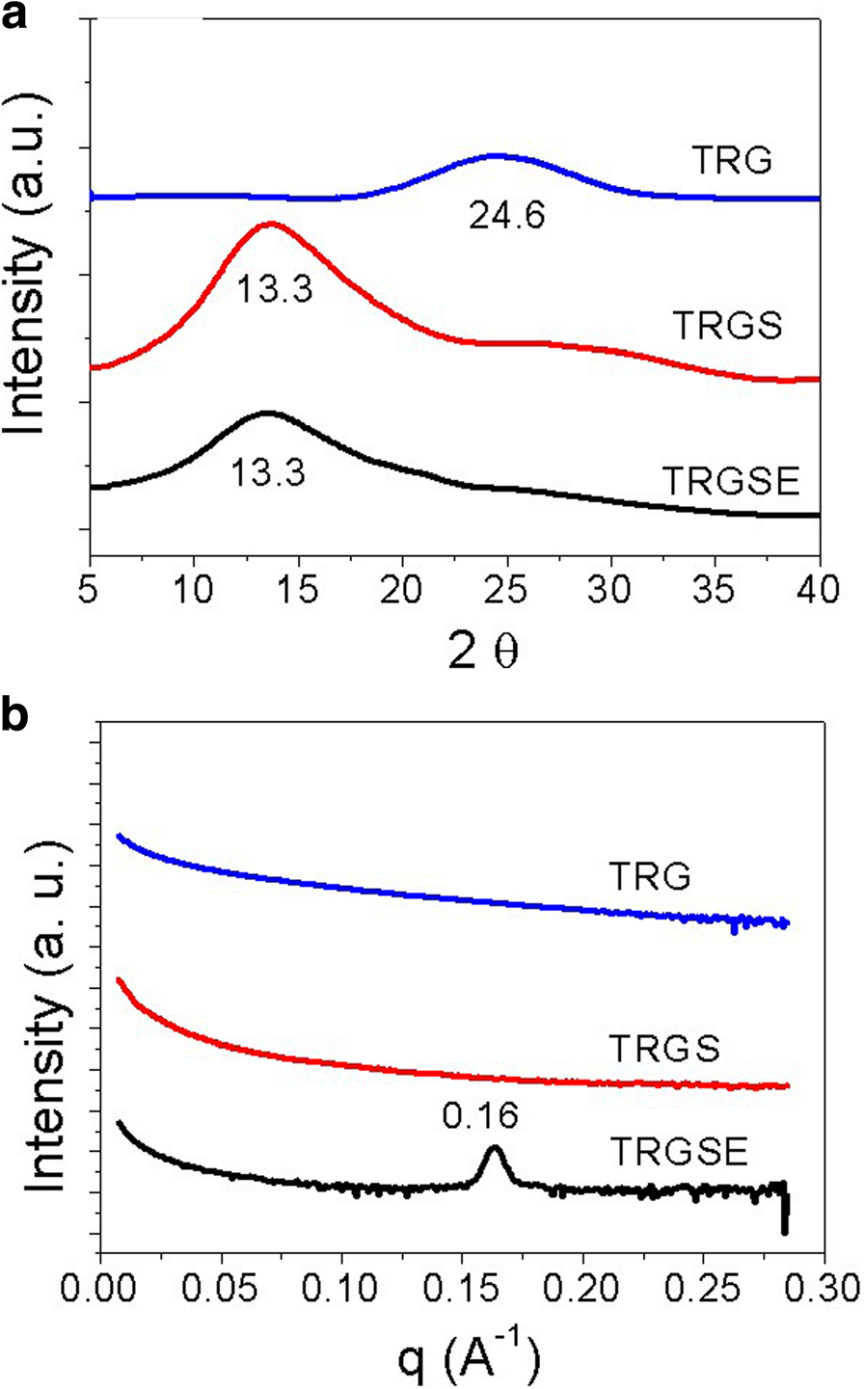
**a** The X-ray diffraction scattering and **b** the small-angle X-ray scattering of the TRG, TRGS, and TRGSE composites

The specific feature of the TRG, TRGS, and TRGSE are revealed by X-ray diffraction (XRD) and small-angle X-ray scattering (SAXS) measurements. Figure 5a displays the X-ray intensity versus the scattering angle 2ɵ. As can be seen, the XRD pattern for TRG shows (001) reflection peak at 24.6° corresponding to the average interlayer distance of 0.36 nm. The TRGS exhibits (001) reflection peak mainly at 13.3°. Thus, the interlayer distance of the TRGS is 0.66 nm. The difference on interlayer distance between TRG and TRGS proved that the SDS is successfully introduced into the interlayer between the TRGS sheets. The interlayer distance of intercalated TRG depends on the size of the intercalated species and the interaction forces [18]. On the other hand, compared with TRGS, the TRGSE has a weaker and broader (001) reflection peak at the same position of 13.3° implying that the interlayer distance of part TRGSE samples has been changed. To confirm the existence of more considerable interlayer distances for these composites at the lower reflection angles, the SAXS measurement was carried out. The SAXS probes the repeating microfeatures in the distance range between few nanometers to few tens of nanometers in polymers or their composites. Figure 5b presented the SAXS pattern obtained from TRG, TRGS, and TRGSE and was expressed as *I* vs. *q* and corrected for background scatter. The repeating structure feature, *d*, of different shapes and sizes, thus can be determined by the scattering vector $q=\frac{4\pi sin\theta}{\lambda }$ in the equation of $d=\frac{2\pi }{q}$ [22]. As can be seen in Fig. 5b, at the low *q* region, the increase in power-law decay exponent represents the scattering of a larger size of objects [22]. For the TRG and TRGS samples, no specific peak is observed over the whole measured region implying that no repeating feature in the distance range of SAXS is detected. However, the TRGSE exhibits a sharp scattering peak at *q* = 0.16 A^−1^ indicating an interlayer distance of 3.92 nm. Compared with the TRGS, this increased interlayer layer distance of TRGSE suggests the formation of SDS/EMI-TFSI complex or micelles.

**Fig. 6:**
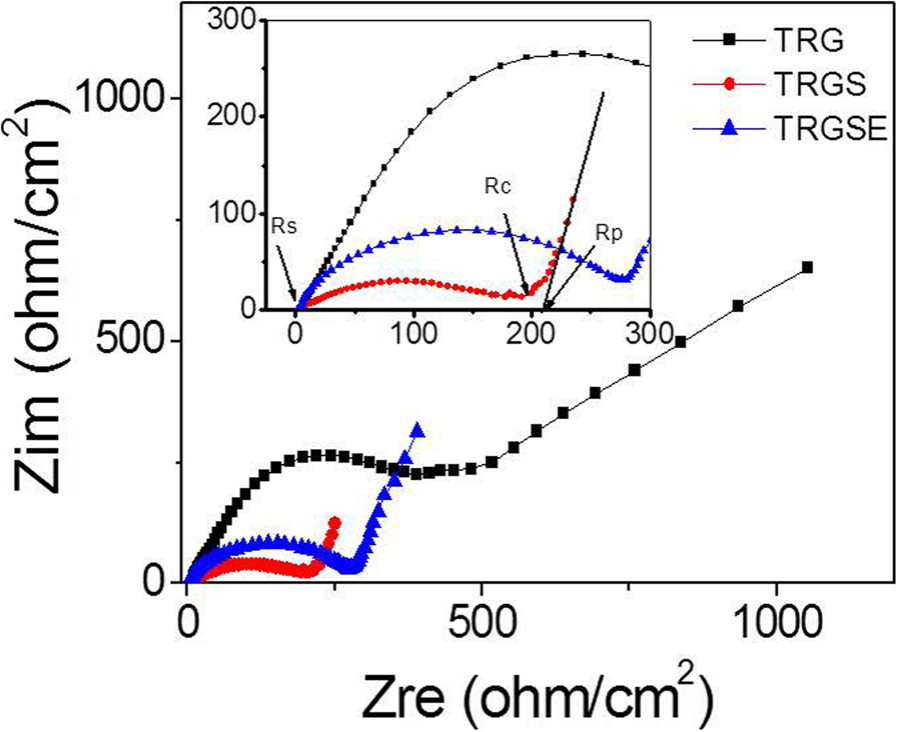
The impedance spectra of the TRG, TRGS, and TRGSE EDLC cells

Figure 6 shows the Nyquist plot for the TRG, TRGS, and TRGSE EDLC cells where the image part of impedance is plotted against the real part of the impedance. From high to low frequency, the impedance curve of TRGS shows a semi-circle followed by a transition zone transferring to a vertical line. As can be seen, the semicircle intercepts or approach the real axis at Rs and Rs + Rc. Rs is usually ascribed to the resistance of ion transport in the electrolyte. In a highly conductive electrolyte, Rc is mainly attributed to the electron conduction in EDLC cells including the contact resistance between graphene particles and that between the graphene electrodes and the current collectors [8, 23]. The high to medium frequency region denotes the charge transfer resistance associated with the porous structure of the electrodes. As observed in TRGS, because of the ion diffusion mechanism between Warburg diffusion and ideal capacitive ion diffusion, the deviation from the vertical line is commonly showing an inclined angle between 45 and 90 against the real axis [23, 24]. This non-ideal capacitance response can be attributed to the pore size distribution that induced different penetration depths of the electrode [24]. The resistance Rp represents the Warburg-related diffusion process and can be approximately deduced by extrapolating the low-frequency data to the real axis. The *x*-axis intercept is therefore equal to the internal resistant *R* = Rs + Rc + Rp [25, 26] as shown in Fig. 6 inset. All the samples have similar Rs values close to 2.8 Ω/cm^2^. The Rc values of the TRG, TRGS, and TRGSE are 388.2, 198.5, and 271.3 Ω/cm^2^, correspondingly. Interestingly, the TRG exhibits the highest Rc of 388.2 Ω/cm^2^. This might be due to the apparent precipitation of TRG aggregates during the preparation of the TRG electrodes. In contrast, the Rc values of both TRGS and TRGSE are relatively smaller than those of the TRG samples. As expected, the TRGSE has a larger resistance than TRG. Because the interlayer distance of TRGSE (3.92 nm) is larger than that of the TRGS (0.66 nm) and this might cause the loose of electrical contact between the TRGSE sheets. On the other hand, at the low-frequency region, the TRG cell shows a clear inclined straight line. This might be due to its small interlayer distance that limited the ion diffusion. Besides, it is found that the Rp of TRGSE (11.2 Ω/cm^2^) is smaller than that of TRGS (21.3 Ω/cm^2^) suggesting that the larger interlayer distance might assist the ion diffusion in between layers. Because the internal resistant *R* is a combination of Rs, Rc, and Rp, the TRGSE (285.3 Ω/cm^2^) cell shows a larger internal resistance *R* than the TRGS (222.6 Ω/cm^2^) cell. These results suggest that the increased interlayer distance could assist ion diffusion but also could reduce the electrical contact between graphene sheets.

**Fig. 7:**
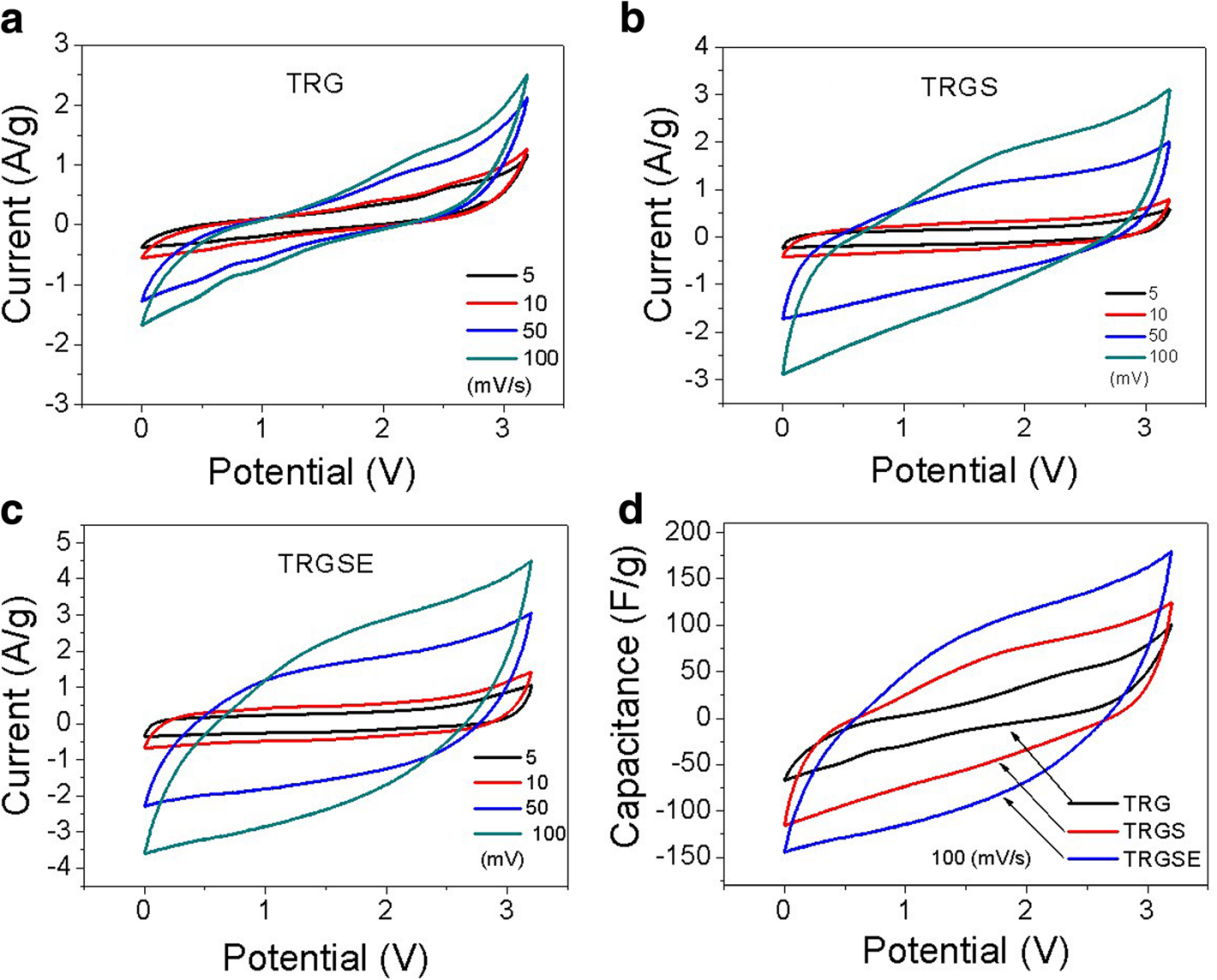
Cyclic voltammetry (CV) curves of **a** TRG, **b** TRGS, and **c** TRGSE cells at the various voltage scan rate and **d** the specific capacitance Cs response of the cells calculated following Cs = (4 × *I*)/(*d* V/d t × m); *d* V/dt is the voltage scan rate, and *m* is the total weight of active materials on two electrodes

Figure 7 plots the cyclic voltammetry (CV) curves of TRG, TRGS, and TRGSE cells. The cells’ current are measured in response to the applied voltage (from 0 to 3.2 V) at various voltage scan rates. The accumulated charge *Q* and the applied voltage *V* follows the *Q* = *CV*, where C is the capacitance of the cell. The response current of the cell follows *I*=*C* × dV/dt. For an ideal capacitance, under the constant voltage scan rate, a constant current should be obtained resulting in a rectangular shape of the CV curve. However, it is considered that the real capacitor is usually in series with an equivalent internal resistor of *R* = Rs + Rc + Rp as discussed earlier. Thus, the charging or discharging current of the capacitor requires a time constant of RC to reach the steady state current [27]. With the increase of RC, it takes more time to reach the steady state and thus collapses the rectangular current profile [28, 29]. As can be seen in Fig. 7a–c, the TRG has a CV curve most seriously deviated from the rectangular curve indicating that TRG has the highest RC time constant which is consistent with its highest internal resistance *R*. On the other hand, increasing the scan rate means reducing the response time and that could also cause the collapsing results as observed in Fig. 7a–c. Figure 7d plots the specific capacitance against voltage profiles in which the CV current at 100 mV/s is divided by the same scan rate. As can be seen, except the TRG, both TRGS and TRGSE cells show a more rectangle capacitance response indicating that they are more close to ideal capacitors. The results indicate the specific capacitance values of the composites are in the sequence of TRGSE>TRGS>TRG.

**Fig. 8:**
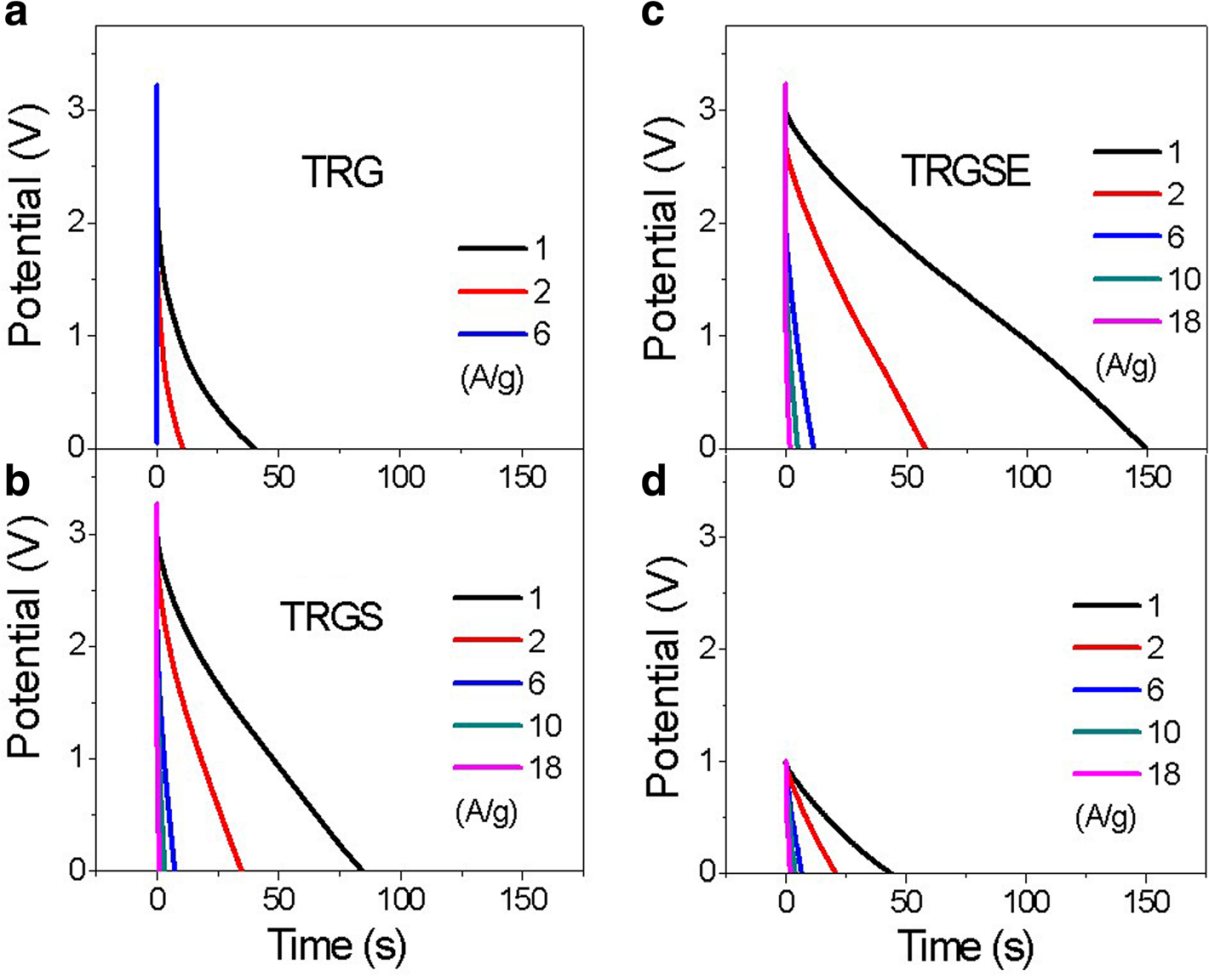
The galvanostatic discharge response vs. time for the **a** TRG, **b** TRGS, and **c** TRGSE cells using IL electrolyte, and **d** the TRGSE cell using 2 M H_2_SO_4_ as electrolyte

**Fig. 9:**
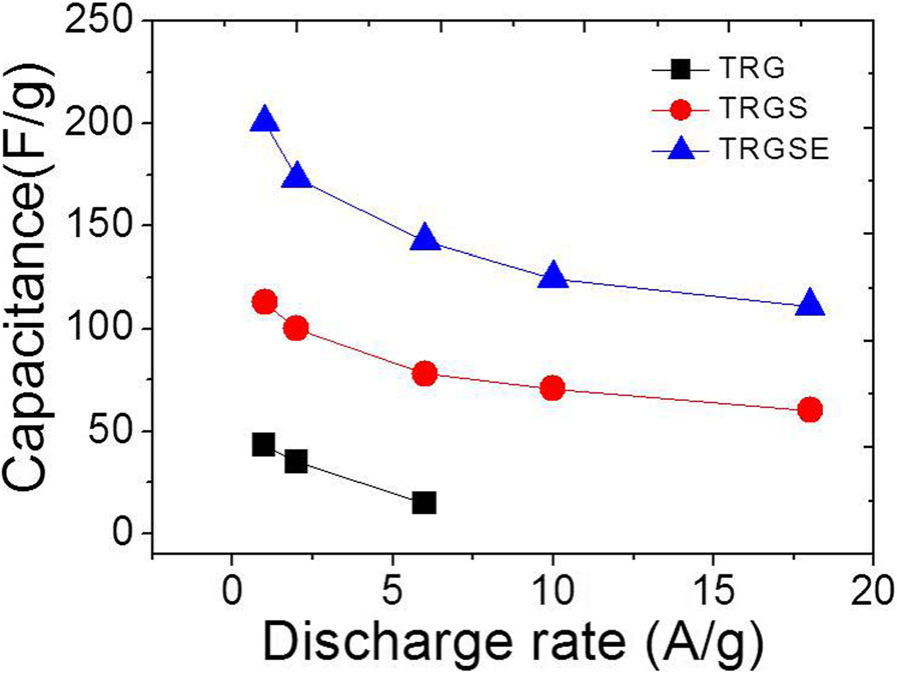
The specific capacitance response of TRG, TRGS, and TRGSE cells at various discharging rates

Due to the wide electrochemical window of EMI-TFSI electrolyte, all the cells can be operated at 3.2 V. The galvanostatic discharge response vs. time of the TRG, TRGS, and TRGSE is plotted in Fig. 8. A voltage drop at the beginning of the discharging current is shown for all the cells. This voltage drop is a result of the voltage loss when the current across an equivalent resistance is related to an internal resistance *R*. Therefore, as observed, the TRG has the highest voltage drop among all the samples. The specific capacitance Cs of the active materials on the electrodes can be extracted from the discharge curve following [7, 30, 31].

$$ {C}_S=\frac{4C}{m}=\frac{4I\Delta t}{m\Delta V} $$

where *m* is the total weight of active materials on two electrodes, *C* is the capacitance of the cell, *I* is the constant current, Δ*t* is the discharging time, and Δ*V* is the potential change (excluding the initial voltage drop) during the discharging. As shown in Fig. 9, the capacitance response decreases as a function of the current density. The decrease in capacitance is because, at high current density, the ions may not have sufficient time to diffuse into the deep region of the pores and tend to accumulate on the surface of the electrodes leading to the decease of accessible surface area and hence capacitance [7]. Therefore, the value of the specific capacitance implies the accessible surface area at the specific current density. At a low current density (1 A/g), the specific capacitances of the TRG, TRGS, and TRGSE are 43.1, 112.6, and 200.5 F/g, respectively. The TRGSE has the highest capacitance which is 1.78 folds higher than that of TRGS indicating the increase of accessible surface area for the TRGSE cell. As expected, at a high current density, the capacitance values of the TRG, TRGS, and TRGSE are decreased to 14.6 F/g (at 6 A/g), 60.2 F/g (at 18 A/g), and 111.1 F/g (at 18 A/g). Still, the TRGSE keep the highest capacitance as the merit of its large interlayer distance that facilitates the ion transport.

The energy densities are obtained by the following equation [7, 30, 31].

$$ E=1/2\ {\mathrm{CV}}^2=1/8\ \mathrm{Cs}\ {\mathrm{V}}^2 $$

And the power density *P* is deduced according to [7, 31].

$$ P=\frac{E}{\Delta t} $$

**Fig. 10:**
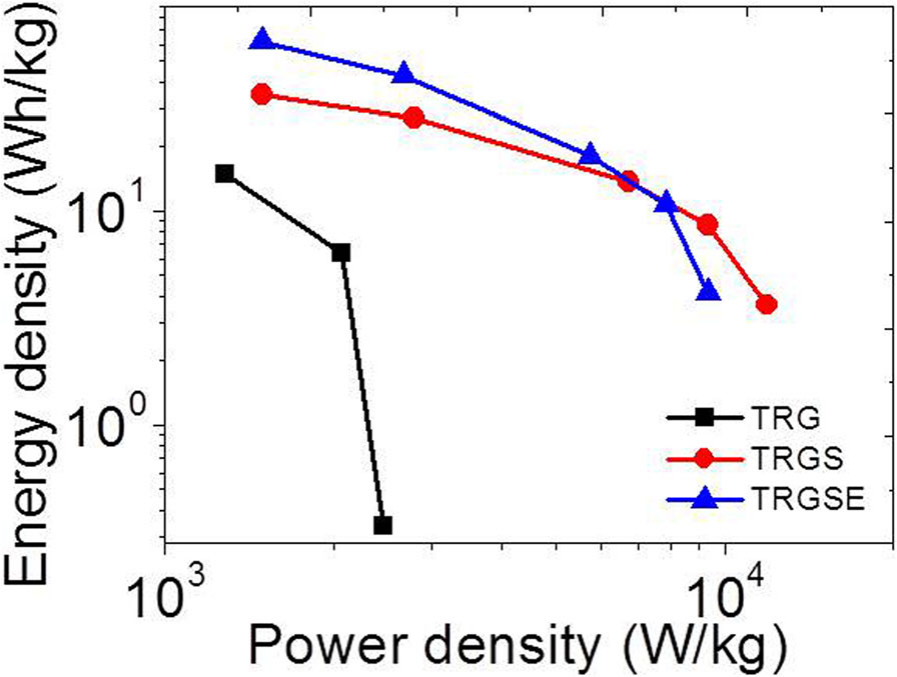
The Ragone plot of the TRG, TRGS and TRGSE cells

Ragone plot (Fig. 10) reveals the energy density of the TRG, TRGS, and TRGSE as a function of power density. Usually, the energy density drops with power density as a result of the voltage decay and capacitance reduction. As can be seen in Fig. 8 and Fig. 9, the large initial voltage drop and the small capacitance of the TRG cell result in its low energy density of 15.3 Wh/kg at 1 A/g. At a low current density (1 A/g), the TRGSE (61.8 Wh/kg) has 1.77 folds higher energy density than TRGS (34.9 Wh/kg). Considering both TRGS and TRGSE have similar initial voltage drops (Fig. 8), the increased energy density of the TRGSE cell would be mainly attributed to its higher capacitance induced by its larger interlayer distance as discussed above. On the other hand, at a high current density, initial voltage drop becomes prominent as the massive current across the equivalent resistor. Due to its large internal resistance and small capacitance, the energy density of the TRG cell is limited at 0.34 Wh/kg (6 A/g). In contrast, at a very high discharging current of 18 A/g, the TRGS and TRGSE still retain the energy density of 3.6 and 4.1 Wh/kg, respectively. For comparison, the discharging response of the TRGSE cell using 2 M of H_2_SO_4_ aqueous solution as electrolyte is also shown in Fig. 8d. At the current density of 1 A/g, the aqueous cell has the specific capacitance of 184.2 F/g. However, due to the small electrochemical window of the aqueous cell, the energy density of the aqueous cell (5.8 Wh/kg) is much smaller than that of the ionic liquid cell (61.8 Wh/kg).

## Conclusion

The influence of the interlayer distance on the performance of the EDLC cells was systematically investigated. The experiment results indicated that the intercalation of SDS surfactant in TRG sheets could prevent the restacking of the TRGS sheets resulting in an interlayer distance of 0.66 nm. A facile approach was demonstrated to tuning the interlayer distance of TRGSE by introducing the EMI-TFSI to interact with TRGS. It is found that the vibration mode of the intercalated SDS is shifted by the interaction with EMI-TFSI IL implying the occurrence of Coulomb interaction between the SDS and EMI-TFSI. It is also found, the interlayer distance of the TRGSE is enlarged to 3.92 nm. These results suggest the formation of large ionic aggregates or micelle in TRGSE sheets. Also, with the large interlayer distance, the TRGSE (200.5 F/g) has a capacitance of 1.78 folds higher than the TRGS (112.7 F/g). Also, the energy density of the TRGSE (61.8 Wh/g) is 1.77 folds higher than that of TRGS (34.9 Wh/kg). The increase in both capacitance and energy density would be attributed to the increased interlayer distance of TRGSE that increase the accessible surface area for the EMI-TFSI electrolyte.

## Methods

**Fig. 11:**
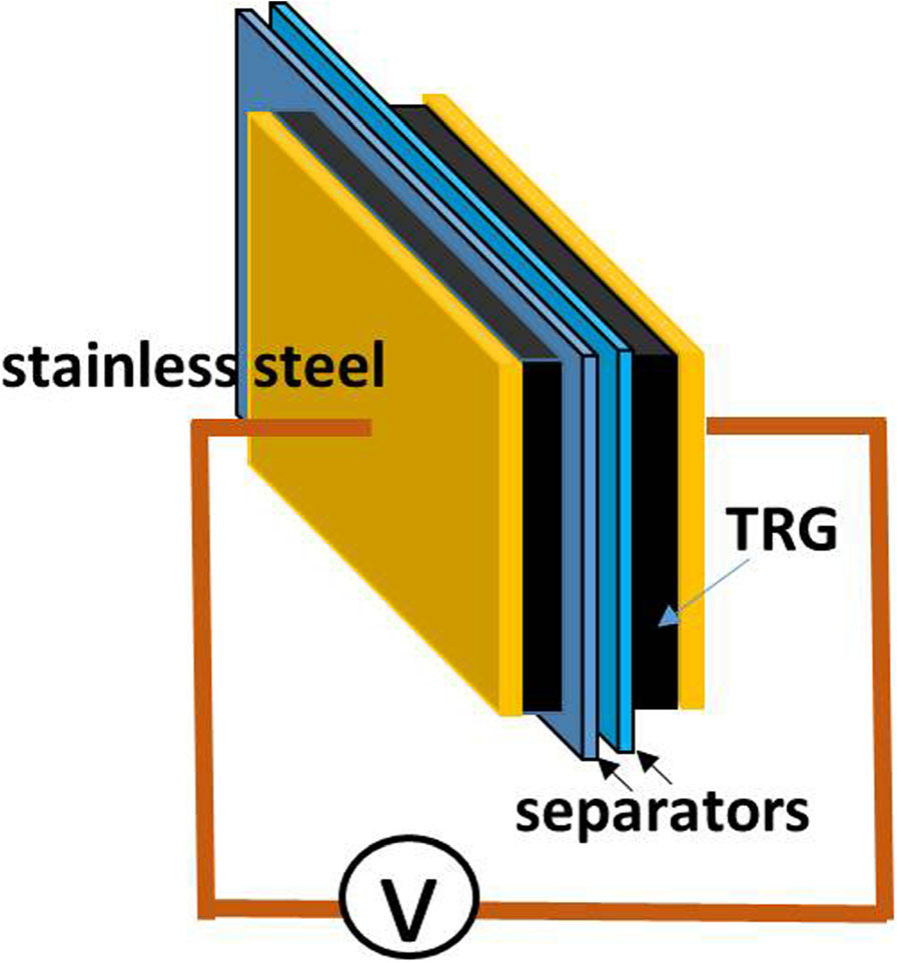
The schematic structure of the EDLC cell

The thermally reduced graphene oxide (TRG) was purchased from GIBusiness company, Taiwan, and was synthesized from natural graphite by a modified Hummers method [11] and followed by the thermal treatment at elevated temperatures. TRGS electrodes are obtained by dispersing 10 mg of TRG powder in 30 ml of 0.1 M SDS solution with the aid of ultrasonication and vigorous stirring for 12 h. After that, the TRGS solution is deposited on a Celgard 3500 separator by a vacuum filter. Further, the TRGS electrodes are rinsed with 15 ml of 0.2 M of EMI-TFSI ethanol solution during filtration to obtain the TRGSE electrodes. For comparison, the TRG electrodes are also produced by dispersing 10 mg of TRG in 20 ml of 20 wt% ethanol solution with the same dispersion and filtration process. The deposited electrode is flipped over on a 1 cm^2^ of 304 stainless steel current collector. The EDLC cell was in the form of a two-electrode package in a sealed testing bag filled with EMI-TFSI as electrolytes for electrical testing using a VersaSTAT 4 potentiostat as shown in Fig. 11. The microstructures of the composites were characterized by the scanning electron microscope (SEM; JEOL-6700, 5 kV), X-ray diffraction (XRD; Bruker-AXS D8, with copper K alpha line, CuKα = 1.5406 A), and small-angle x-ray (SAXS; Nanostar U system, Bruker AXS Gmbh, CuKα = 1.5406 A). The weight composition was determined by the thermal gravimetric analysis (TGA; TA Instruments, TA Q50), at a heating rate of 10 °C/min under nitrogen flow, and. Fourier-transform infrared spectrometer (FTIR; Bruker Vertex 70v) was also carried out to investigate the bond vibration in a wave number range of 4000 to 400 cm^−1^.

## Abbreviations

EDLC: Electrical double layer capacitor
EMI-TFSI: 1-Ethyl-3-methylimidazolium bis(trifluoromethylsulfonyl)imide
FTIR: Fourier-transform infrared spectroscopy
GO: Graphene oxide
RGO: Reduced graphene oxide
SAXS: Small angle X-ray scattering
SDS: Sodium dodecyl sulfate
SEM: Scanning electron microscope
TGA: Thermal gravimetric analysis
TRG: Thermally reduced graphene oxide
TRGS: SDS-intercalated TRG composite
TRGSE: TRGS are rinsed with the EMI-TFSI solution during the filtration
XRD: X-ray diffraction
